# Nanoparticles Used for the Delivery of RNAi-Based Therapeutics

**DOI:** 10.3390/pharmaceutics17111502

**Published:** 2025-11-20

**Authors:** Tianrui Ren, Liang Ma, Ping Fu, Chuyue Zhang

**Affiliations:** Department of Nephrology, Kidney Research Institute, Science & Technology Department, West China Hospital of Sichuan University, Chengdu 610041, China

**Keywords:** liposomes, polymeric nanoparticles, exosomes, miRNA, siRNA

## Abstract

RNA interference (RNAi) offers programmable, sequence-specific silencing via small interfering RNA (siRNA) and microRNA (miRNA), but clinical translation hinges on overcoming instability, immunogenicity, and inefficient endosomal escape. This review synthesizes advances in non-viral nanocarriers—liposomes, polymeric nanoparticles, and extracellular vesicles (EVs)—that stabilize nucleic acids, tune biodistribution, and enable organ- and cell-selective delivery. We highlight design levers that now define the field: ligand-guided targeting, stimuli-responsive release, biomimicry and endogenous carriers, and rational co-delivery with small molecules. Across major disease areas—cancer and cardiovascular, respiratory, and urological disorders—these platforms achieve tissue-selective uptake (e.g., macrophages, endothelium, and myocardium), traverse physiological barriers (including the blood–brain barrier and fibrotic stroma), and remodel hostile microenvironments or immune programs to enhance efficacy while maintaining favorable safety profiles. Early clinical studies reflect this diversity, spanning targeted nanoparticles, local drug depots, exosome and cellular carriers, and inhaled formulations, e.g., and converge on core phase-I endpoints (safety, maximum tolerated dose, pharmacokinetics/pharmacodynamics, and early activity). Looking ahead, priorities include good manufacturing practice scale, consistent manufacture—especially for EVs; more efficient loading and cargo control; improved endosomal escape and biodistribution; and rigorous, long-term safety evaluation with standardized, head-to-head benchmarking. Emerging directions such as in vivo EVs biogenesis, theragnostic integration, and data-driven formulation discovery are poised to accelerate translation. Collectively, nanoparticle-enabled RNAi has matured into a versatile, clinically relevant toolkit for precise gene silencing, positioning the field to deliver next-generation therapies across diverse indications.

## 1. Introduction

RNA interference (RNAi) was first discovered by Fire et al. in 1998 [1] and is an evolutionarily conserved gene-regulatory mechanism mediated by exogenous double-stranded RNA (dsRNA) that triggers sequence-specific, post-transcriptional gene silencing. It works through the degradation of specific mRNA, thereby preventing its translation into proteins [2]. The mechanism relies on two classes of small RNA molecules: microRNA (miRNA) and small interfering RNA (siRNA). These RNA molecules achieve gene silencing by binding to specific mRNAs through complementary base pairing, either by degrading the mRNA or inhibiting its translation. miRNAs are endogenous, non-coding small RNAs of about 18–25 nucleotides in length that act as molecular switches to regulate gene expression post-transcriptionally. By specifically base-pairing with the mRNAs of protein-coding genes in animals and plants, they induce translational repression or promote mRNA degradation, thereby precisely modulating gene-expression networks in recipient cells [3,4]. siRNA is a double-stranded RNA molecule of approximately 20–25 nucleotides in length, with two unpaired nucleotides at the 3′ end of each strand. It plays a central role through RNAi pathway by hybridizing with complementary mRNA molecules to guide the RNA-induced silencing complex (RISC) to specifically degrade target mRNAs, thereby silencing the expression of specific genes [5]. Therefore, miRNAs and siRNAs share the same core RNAi mechanism but differ in origin and targeting breadth, enabling both physiological regulation and experimental/therapeutic manipulation. Exogenous siRNA duplexes and miRNA mimics/inhibitors harness RNAi to probe gene function and offer clinical potential for precise, programmable gene silencing.

However, RNA delivery, particularly for siRNA and miRNA, still faces multiple challenges that limit their therapeutic potential. The main obstacles include stability, immunogenicity and delivery efficiency. Firstly, naked nucleic acids have poor in vivo stability and are rapidly degraded in the bloodstream. Its relatively high molecular weight, negative charge, and hydrophilicity hinder translocation across cell membranes. They tend to accumulate in the kidneys and be excreted in urine, or be captured by the reticuloendothelial system, preventing effective binding to target sequences and thus limiting its functional activity [6]. Secondly, the introduction of foreign nanomaterials into the bloodstream can trigger a cascade of innate immune responses, potentially compromising both safety and efficacy. Two primary concerns are complement activation and the induction of inflammatory cytokine responses, which can be triggered by both the nanoparticle carrier and its nucleic acid payload. Complement activation is an immediate, antibody-independent response often initiated upon intravenous administration. The surfaces of many nanoparticles can activate the complement system, leading to opsonization with fragments like C3b, which marks the particle for rapid clearance by the reticuloendothelial system (RES) [7]. This not only curtails circulation half-life but can also generate potent anaphylatoxins (C3a, C5a), causing infusion-related reactions, sometimes termed complement activation-related pseudoallergy (CARPA) [8]. A widely adopted strategy to mitigate this is PEGylation, which creates a “stealth” layer to hinder protein adsorption and prolong circulation [9]. Another major concern is the pro-inflammatory cytokine response, which is largely driven by the recognition of the RNAi payload itself. The innate immune system can recognize exogenous RNA via pattern recognition receptors (PRRs) like endosomal Toll-like receptors (TLR3, TLR7, TLR8) and cytosolic sensors like retinoic acid-inducible gene I (RIG-I) and melanoma differentiation-associated gene 5 (MDA5) [10]. Activation of these pathways triggers a “cytokine storm” (e.g., Type I interferons, TNF-α), leading to systemic inflammation and potential off-target effects. Significant progress has been made to abrogate this response through chemical modifications of the RNA backbone (e.g., 2′-O-methyl), which effectively shield it from PRR recognition without compromising silencing activity [11]. Thirdly, delivery efficiency of RNA-based therapeutics is limited by intracellular trafficking, especially endosomal escape [12]. After endocytic uptake, non-viral oligonucleotides are sequestered in early endosomes that mature into late endosomes and ultimately fuse with lysosomes enriched with degradative enzymes. Unless these cargos escape to the cytosol before lysosomal fusion, they are degraded and cannot engage the RNAi machinery, resulting in reduced therapeutic efficacy and elevated off-target toxicity [13,14]. Due to these challenges of stability, immunogenicity, and cellular delivery, precise delivery to target tissues has become a central objective in the research and development of nucleic acid drugs (NADs). Consequently, substantial advances in nanoparticle technologies and delivery vehicles, designed to overcome these specific hurdles, have markedly improved delivery efficiency and form the basis of this review.

## 2. Methods: Search Strategy and Selection Criteria

This narrative review was compiled following a structured literature search of the PubMed database for relevant articles published between 1 January 2021, and 31 August 2025. The search strategy was designed to identify studies on nanoparticle-mediated delivery of RNAi-based therapeutics. Specific keywords were combined to retrieve articles for distinct nanoparticle platforms. For liposomal systems, the search query was: (liposomes [Title]) AND ((miRNA [Title/Abstract]) OR (siRNA [Title/Abstract])). For polymeric nanoparticles, the query was: (Polymeric Nanoparticles [Title/Abstract]) AND ((siRNA [Title/Abstract]) OR (miRNA [Title/Abstract])). For exosomes and extracellular vesicles, the search was conducted using: ((Exosomes [Title]) OR (extracellular vesicles [Title])) AND ((miRNA [Title]) OR (siRNA [Title])). Inclusion criteria: (1) Studies involving the treatment of solid tumors/neoplasms, and clinical or animal studies related to cardiovascular diseases, respiratory diseases, or urinary system diseases; (2) Publications in journals with an impact factor of ≥5. Exclusion criteria: Antisense oligonucleotides (ASOs), aptamers, mRNA-based therapeutics, and RNA editing (CRISPR).

## 3. Nanocarriers for Delivering RNAi-Based Therapeutics

Currently, a broad range of nanocarriers have been developed for siRNA, miRNA deliver into tissues, addressing key in vivo barriers. The principal platforms are lipid-based nanoparticles (e.g., liposomes, micelles, solid lipid nanoparticles), polymer-based nanoparticles (e.g., chitosan, dendrimers, protein-based carriers) and extracellular vesicles (e.g., exosomes, micro vesicles, apoptotic bodies). This review focuses on organ-targeted therapeutic advances achieved with three representative platforms—liposomes, polymeric nanoparticles, and exosomes. Because siRNA and miRNA are structurally similar, they often employ the same formulations to enhance stability and improve in vivo pharmacokinetics. siRNAs, being rigid, double-stranded duplexes (≈21–23 bp), possess a high charge density, which facilitates more efficient loading (typically 10–30% *w*/*w* in lipid nanoparticles) and greater stability. In contrast, miRNA mimics, which are often single-stranded and more flexible (≈19–25 nt), have a lower charge density, resulting in lower loading efficiency (often 5–15% *w*/*w* in lipid nanoparticles) and a higher propensity for premature release. This divergence necessitates tailored nanocarrier design, where formulations for miRNAs may require chemical modifications to improve loading, while the high efficiency of siRNA loading allows for optimization strategies to minimize potential off-target effects. Accordingly, this section focuses on the characteristics of these non-viral RNAi delivery systems, with particular emphasis on organ-specific targeting strategies.

### 3.1. Liposomes

While “lipid nanoparticles” (LNPs) is a broad term that technically includes conventional liposomes, in modern scientific literature, LNPs refer to a distinct class of delivery vehicles optimized for nucleic acids. Conventional liposomes are colloidal, spherical vesicles formed by self-assembly of amphiphilic lipids in aqueous media, with polar headgroups facing the inner and outer aqueous phases and hydrophobic tails forming bilayers around an aqueous core [15]. Their classification is based on lamellarity and size, including small unilamellar vesicles (SUVs, <100 nm), large unilamellar vesicles (LUVs, 100–500 nm), giant unilamellar vesicles (GUVs, >500 nm), oligolamellar vesicles (OLVs, 2–5 bilayers), multilamellar vesicles (MLVs, >5 bilayers), and multivesicular vesicles (MVVs) with internal compartments [16,17]. However, recent advancements have pushed design principles far beyond simple modulation. Modern strategies now focus on creating “smart” liposomes with sophisticated functionalities. These include stimulus-responsive systems engineered to release payloads upon exposure to physical triggers like ultrasound for site-specific delivery [18], advanced surface modifications such as one-step self-assembly of chimeric nanobodies to create immunoliposomes with high targeting efficiency [19], and multi-functional biomimetic designs. For example, liposomes can be coated with red blood cell membranes for immune evasion and conjugated with ligands like hyaluronic acid to specifically target pro-inflammatory macrophages in atherosclerotic plaques [20]. Furthermore, highly complex, dual-functionalized liposomes are being developed that combine neuron-targeting peptides (e.g., RVG29) with reactive oxygen species (ROS)-responsive elements to cross the blood–brain barrier (BBB) and achieve precise, stimulus-gated drug release in ischemic brain tissue [21]. In contrast, modern LNPs, which dominate the RNAi and mRNA delivery landscape, possess a different structure and composition. Instead of a distinct aqueous core, LNPs feature a dense, electron-rich core where nucleic acids are complexed with ionizable cationic lipids. This LNP framework allows for sophisticated chemical innovation. For instance, advanced LNPs have been engineered with phenylboronic acid (PBA)-modified lipids that target sialic acid on melanoma cells and trigger adenosine triphosphate (ATP)-responsive intracellular siRNA release, significantly boosting antitumor effects [22]. Furthermore, to overcome off-target liver accumulation, novel peptide-ionizable lipids have been rationally designed, enabling the creation of LNPs with tissue-specific tropism for organs like the lungs, spleen, and bone, and even facilitating in vivo prime editing [23]. Liposomes can encapsulate and protect RNAi molecules, achieve targeting via structural modification to enhance their cellular uptake and improve their biodistribution, and also reduce off-target toxicity, making them efficient carriers suitable for the delivery of RNAi-based therapeutics. While both platforms protect RNAi molecules from degradation and can be engineered for targeted delivery, their primary applications diverge. Conventional liposomes remain a versatile platform, particularly for approved drugs carrying small-molecule payloads. However, for RNAi-based therapeutics, LNPs are the preferred and more advanced system due to their specialized structure that efficiently encapsulates, protects, and facilitates the intracellular delivery of siRNA and miRNA [24].

### 3.2. Polymeric Nanoparticles

Polymeric nanoparticles are nanoscale carriers (from 1 nm to 1000 nm) constructed from natural or synthetic polymers. They differ from inorganic and lipid systems by offering finely tunable size, shape, surface charge, and a broad range of chemical and biological functionalities [25]. These features support targeted delivery to tissues and subcellular compartments and enable stimulus-responsive release, improving efficacy across diverse cargos [26]. Within this class, functional categories include drug-loaded systems (e.g., albumin-bound formulations, polymeric micelles) and optical probes such as dye-loaded polymeric nanoparticles and semiconducting polymeric nanoparticles, which provide high brightness, photostability, and spectral tunability [27]. Owing to the advantages of polymeric nanoparticles, nucleic acids (including RNAi, the focus of this review) can achieve enhanced stability and site-specific intracellular delivery.

### 3.3. Extracellular Vesicle

Exosomes are small extracellular vesicles (40–160 nm) of endosomal origin that carry DNA, RNA, lipids, metabolites, and proteins reflective of their parent cells [28]. EVs are broadly classified into ectosomes, plasma membrane, derived vesicles (~50 nm to 1 μm), and exosomes, which arise from multivesicular bodies [29]. Exosomes have emerged as versatile delivery platforms capable of transporting functional cargo (e.g., miRNA, siRNA) that can promote or restrain disease processes across immunology, infectious diseases, pregnancy-related conditions, cardiovascular, kidney repair [30,31,32], and cancer [33,34]. As drug carriers, exosomes offer high biocompatibility, low toxicity, deep tissue penetration, broad dissemination via the bloodstream, the ability to cross the blood–brain barrier, and the capacity to bypass P-glycoprotein efflux [35]. Key design strategies involve engineering exosomes to package and deliver defined payloads to specific cell types or tissues. Mechanistically, their lipid bilayer and tetraspanin-rich membranes shield RNA cargos from serum nucleases and complement, while endogenous surface cues (e.g., integrins, CD47) support immune evasion, prolonged circulation, and organ tropism [36]. Taken together, these attributes explain why EVs are effective carriers for RNAi: they provide nuclease protection, targeted biodistribution, and improved cytosolic access, resulting in enhanced stability and site-specific intracellular delivery of nucleic acids, including the RNAi therapeutics central to this review.

## 4. Nanoparticles Used for the Delivery of RNAi-Based Therapeutics Against Cancer

### 4.1. siRNA-Based Cancer Therapeutics

#### 4.1.1. siRNA-Based Cancer Therapeutics via Liposomes

Liposomal platforms are accelerating siRNA therapeutics across diverse malignancies by uniting ligand-guided targeting, biomimicry, stimuli-responsiveness, and combinatorial payloading to address circulation, penetration, and intracellular release barriers. In lung cancer, an alginate hydrogel embedding endoplasmic reticulum (ER)-modified liposomes co-delivering STAT3 siRNA and lidocaine achieved single-application tumor control, tumor-associated macrophage (TAM) repolarization, natural killer cell (NK) activation, pleural effusion reduction, and analgesia in postoperative non-small cell lung cancer (NSCLC) models, exemplifying local immunoanalgesic management [37], while a focused review delineates formulation advances and translational hurdles specific to siRNA-loaded liposomes in this disease [17]. In hepatocellular carcinoma, tLyP-1–modified liposomes co-delivering SN38 and MEF2D siRNA activated cGAS–STING yet reversed PD-L1 upregulation, reduced M2/MDSCs, expanded intratumoral CD4+ T cells, and suppressed tumor growth [38]; sorafenib plus KIAA1199 siRNA co-loaded liposomes synergistically curtailed proliferation, migration, and invasion with favorable biodistribution and safety [39]; ultrasound-sensitive liposomes released sonosensitizer and siBcl-2 upon insonation, using ROS to rupture vesicles and enable lysosomal escape for gene silencing [40]; and a macrophage-membrane–cloaked, cRGD/CPP-decorated thermosensitive system achieved HepG2 selectivity, strong *Bcl-2* knockdown, minimal RES uptake, and robust antitumor effects under mild hyperthermia [41]. For brain tumors, polymer-locking fusogenic liposomes (Plofsomes) crossed the BBB and fused in ROS-rich glioblastoma (GBM) to deliver siRNA or CRISPR ribonucleoprotein complexes (RNPs), suppressing midkine to reduce temozolomide resistance with tumor-restricted activity [42], while echogenic liposomes served as cavitation nuclei for sonoporation in vivo, supporting a physical-trigger route adaptable to siRNA delivery [43]. In breast cancer, brown rice bran powder1 (BRBP1)-modified liposomes traversed the BBB to brain metastases, co-delivering paclitaxel and TWF1 siRNA to reverse epithelial-mesenchymal transition (EMT)-linked taxane resistance and inhibit metastatic growth [44]; cationic liposomes co-loaded crizotinib, paclitaxel, and Bcl-xL siRNA provided coordinated release, synergistic cytotoxicity, and gene knockdown in MCF-7 cells [45]; and Herceptin-conjugated liposomes carrying Lipocalin-2 (LCN2) siRNA internalized efficiently into HER2+ inflammatory breast cancer cells, reduced LCN2 and disrupted tumor emboli, reshaping oncogenic pathways [46]. In pancreatic ductal adenocarcinoma, macrophage-mimetic, LFC131-targeted, CaP-cored liposomes silencing glutamine-fructose-6-phosphate aminotransferase 1 (GFAT1) lowered hyaluronan, decompressed vasculature, and weakened stroma; combined with Doxil, they enhanced penetration and antitumor efficacy while reducing invasiveness versus hyaluronidase [47]. When combining siRNA with other modalities, sequence/schedule dependencies and toxicity management become critical design considerations. For example, cationic liposomes co-encapsulating hydrophobic proteolysis targeting chimeras (PROTACs (DT2216)) and siRNA leveraged lipid solubilization and polymeric condensation to amplify protein degradation and tumor inhibition, showcasing dual modality payloading of hydrophilic and hydrophobic agents [48]. Crucially, the toxicity of such dual-payload systems was rigorously evaluated; in vivo studies using H&E staining of major organs (heart, liver, spleen, lungs, and kidneys) from treated subjects confirmed that the liposomal co-delivery vehicle did not induce significant histopathological abnormalities, demonstrating effective toxicity management. Similarly, in combinations of chemotherapeutics and siRNA, schedule dependence is a key determinant of efficacy. Studies pairing paclitaxel (PTX) and a survivin-targeting siRNA within a single cationic liposome formulation revealed a clear synergistic effect. This synergy was schedule-dependent, observed only when the two agents were co-loaded and delivered simultaneously, which produced significantly higher cytotoxicity (*p* < 0.01) compared to their administration as separate solutions. This highlights the necessity of synchronized delivery to distinct intracellular targets to maximize the therapeutic outcome. Collectively, these studies converge on five design levers—ligands (tLyP-1, BRBP1, folate, Herceptin), biomimicry (macrophage membranes), stimuli (thermal, ultrasound, ROS), rational combinations (chemotherapeutics, PROTACs), and microenvironment remodeling (HA downregulation)—to advance precise, safe, and effective liposomal siRNA therapeutics toward clinical translation across cancer types.

#### 4.1.2. siRNA-Based Cancer Therapeutics via Polymeric Nanoparticles

Polymeric nanoparticles (PNPs) are redefining siRNA oncology by coupling modular chemistries, ligand display, and stimuli-responsiveness to stabilize cargo, enable tumor-specific uptake, and promote endosomal escape, complementing lipid systems and expanding delivery beyond the liver [25,49,50,51]. Platform advances include high-throughput poly (ethylene oxide) (PEO)–poly (ε-caprolactone) (PCL)–amine triblock libraries that identify carriers with favorable circulation and tumor accumulation in vivo [52], and Photocrosslinked, Bioreducible designs with ester/disulfide linkages that improve serum stability and cytosolic release to achieve potent knockdown in glioma and melanoma, including lung-colonized lesions after systemic dosing [53]. In lung cancer, anisamide-functionalized oligopeptide end-modified poly (beta aminoester) (OM-pBAE) nanoparticles selectively deliver mTOR siRNA to NSCLC, maintaining stability in serum, achieving efficient transfection, and suppressing tumor growth in vitro and in vivo with tumor selectivity [26]. For hepatocellular carcinoma, expert consensus emphasizes nanoparticle engineering to cross vessel walls and extracellular matrix (ECM) and target hepatocellular carcinoma (HCC) receptors [54], while AFP-siRNA-loaded PLGA (PLGA-AFP) platforms synergize with epigallocatechin gallate to amplify apoptosis [55] and with low-dose sorafenib/sunitinib to deepen AFP silencing and inhibit proliferation, supporting dose-sparing combinations [56]. In breast cancer, especially triple-negative breast cancer (TNBC), dermatan sulfate/chitosan “double self-assembled” NPs leverage CD44 targeting to deliver *BIRC5* (survivin) siRNA and curb proliferation, migration, and spheroid formation [57]; PLGA nanovectors decorated with a TNBC-specific aptamer enable rapid, selective programmed cell death-ligand1 (PD-L1) silencing and endosomal escape in MDA-MB-231/BT-549 cells [58]; smart pH-responsive, PEG-detachable co-delivery of paclitaxel and vascular endothelial growth factor (VEGF) siRNA inhibits angiogenesis and outperforms monotherapies in 4T1 models [59]; and immune-centric mPEG-PLGA-PLL (PEAL) nanoparticles that carry siCD155 and are coated with anti-PD-L1 antibodies orchestrate asynchronous checkpoint blockade, eliciting CD8+ TIL-dominant immunity to suppress TNBC growth and metastasis with safety [60]. In ovarian cancer, an intraperitoneal, thermosensitive hydrogel reservoir provides sustained release of STAT3-siRNA polyplexes, enriches nodules, and significantly delays tumor growth in advanced models, aligning with reviews positioning PNPs to overcome systemic instability and chemoresistance [61,62]. Pancreatic cancer efforts exploit HA-displaying chitosan/siRNA NPs to target CD44 and knock down HIF-1α under hypoxia, with chitosan molecular weight tuning uptake versus silencing and in vivo gene suppression [63], complemented by broader evidence for siRNA-NP co-therapies and translational momentum [64], and by cholesterol-modified PNPs delivering Inhba siRNA to suppress activin A, reducing Pancreatic ductal adenocarcinoma (PDAC) growth/metastasis and cachexia while reshaping the tumor microenvironment (TME) [65]. Negative design lessons emerge in bladder cancer, where survivin siRNA plus paclitaxel lacked synergy due to survivin-induced cell-cycle arrest antagonizing taxanes, underscoring schedule/biology-aware co-delivery [66]. Beyond tumor cells, tyrosine-modified polyethylenimine/polypropylenimine (PEI/PPI) systems efficiently transfect macrophages; siSTAT3/siSTAT6 reprograms tumor-associated macrophages (TAMs) toward phagocytic M1 phenotypes and enhances tumor cell clearance [67]. Finally, lipid-assisted mPEG-PLGA hybrids optimized for anti-CD47 siRNA demonstrate potent antitumor immunity in melanoma, illustrating immunogene targets enabled by polymer–lipid synergy [68]. Collectively, ligand-guided targeting, redox/pH/Photo-crosslinkable architectures, rational co-delivery, immune reprogramming, and locoregional depots are converging to deliver tumor- and context-tailored siRNA therapies across cancer types.

#### 4.1.3. siRNA-Based Cancer Therapeutics via Exosomes and Other Extracellular Vesicles

EVs are emerging as biocompatible, targetable carriers that overcome key barriers to siRNA therapy by improving stability, biodistribution, immune tolerance, and tumor penetration. Scalable sources such as bovine milk/colostrum exosomes enable high loading, ligand functionalization, and reduced toxicity, positioning them as cost-effective platforms for systemic delivery, including siRNA-based therapeutics [34]. In lung cancer, edible kiwi-derived vesicles loaded with siSTAT3 and decorated with an epidermal growth factor receptor (EGFR) aptamer safely suppressed growth of multidrug-resistant EGFR-mutant NSCLC, highlighting charge-free, plant-derived EVs for precision targeting [69] while an engineered hybrid of EVs with gold–siRNA nanocomplexes silenced the B7-H4 checkpoint, curtailed proliferation/invasion, and achieved antitumor efficacy in NSCLC xenografts with limited effects in normal cells [70]. For brain tumors, blood exosomes co-delivering cytoplasmic phospholipase A2 (*cPLA2*) siRNA and metformin crossed the BBB and reprogrammed glioblastoma energy metabolism in patient-derived xenograft (PDX) models, prolonging survival and enabling biomarker-guided personalization via polymerase 1 and transcript release factor (PTRF)-mediated uptake [71]; macrophage exosomes co-loaded with panobinostat and mutation of p53-induced protein phosphatase 1(PPM1D) siRNA similarly achieved BBB transit and extended survival in diffuse intrinsic pontine gliomas (DIPG) [72]. Tumor and immune microenvironments can be remodeled with EV–siRNA: M1 macrophage–derived exosomes carrying siSIRPα repolarize M2 macrophages and, with anti-PD-L1, enhance phagocytosis and suppress metastatic traits [73]; M1 EVs delivering CX3CR1 siRNA inhibit PDAC proliferation and migration and suppress tumors in vivo [74]. In ovarian cancer, patient-derived exosomes electroporated with c-Met siRNA selectively accumulated in peritoneal lesions and extended survival after intraperitoneal dosing, supporting autologous, surgery-integrated production [75]. In triple-negative breast cancer, EGFR-aptamer-modified exosomes co-delivering delivery of aspartyl-tRNA synthetase-antisense RNA 1 (DARS-AS1) siRNA and doxorubicin overcame autophagy-mediated chemoresistance via TGF-β/Smad3 inhibition [76], while MEK1-siRNA exosomes downregulated mitogen-activated protein kinase/extracellular signal-regulated kinase (MAPK/ERK) signaling and reduced angiogenesis [77]. Collectively, natural-source, engineered, and immune-cell–derived EVs are enabling targeted siRNA therapies across cancer types, with remaining priorities in scalable manufacture, loading efficiency, and long-term safety benchmarking versus synthetic nanocarriers.

### 4.2. miRNA-Based Cancer Therapeutics

#### 4.2.1. miRNA-Based Cancer Therapeutics via Liposomes

Liposomal carriers are enabling cancer type–specific miRNA therapeutics by integrating active targeting, organelle tropism, and theranostic design. In a novel theoretical model, optically guided liposomes are proposed for gene delivery to inhibit enzymes involved in drug metabolism. This strategy aims to overcome chemoresistance, allowing for the effective use of inexpensive, existing chemotherapeutics and potentially reducing treatment costs while improving patient outcomes [78]. In lung cancer, mitochondria-targeted, pH-responsive polymeric liposomes (R@DA-TPP-SA) enabled pre-miR-34a imaging via intracellular hybridization chain reaction (HCR) and mitochondrial delivery of therapeutic miR-34a, achieving lysosomal escape, triphenylphosphine (TPP)-mediated organelle accumulation, loss of membrane potential, apoptosis, and in vivo antitumor efficacy with low toxicity—showcasing an organelle-precise, imaging-guided therapy paradigm [79]. For hepatocellular carcinoma, a focused review underscores lipid nanoparticles as leading non-viral vectors for miRNA/siRNA, detailing advances in tumor-specific targeting and transfection alongside formulation challenges (stability, specificity, immunogenicity) and encouraging preclinical outcomes [80]. Broadly across solid tumors, a theoretical framework proposes optically guided, layered liposomes packaging miRNAs to inhibit drug-metabolizing enzymes, thereby resensitizing resistant cancers to affordable chemotherapies and potentially lowering costs while improving outcomes [78]. Collectively, targeted, stimuli-responsive, and organelle-directed liposomal systems are maturing from concept to translational platforms that enhance delivery fidelity, enable real-time monitoring, and potentiate existing therapies across cancer types.

#### 4.2.2. miRNA-Based Cancer Therapeutics via Polymeric Nanoparticles

Polymeric nanoparticles are accelerating miRNA therapeutics across solid and hematologic malignancies by protecting labile cargos, enabling ligand-guided uptake, and programming on-demand release (for example via redox-responsive chemistries) [25]. In multiple myeloma, reviews highlight polymeric platforms among non-viral nanocarriers that improve tumor targeting and reduce systemic toxicity for miRNA delivery [81]. In glioblastoma, Angiopep-2-decorated PNPs co-deliver anti-miR-21 and miR-124 across the blood–brain barrier to co-regulate RAS/PI3K/PTEN/AKT signaling, suppress invasion/angiogenesis, and extend survival in orthotopic models [82]. For cutaneous squamous cell carcinoma, dual cell-penetrating peptide–conjugated PNPs transporting miR-205-5p achieve superior internalization, G0/G1 arrest, apoptosis, and in vivo tumor volume reduction with minimal off-target toxicity [83]. In pancreatic cancer, PNP-mediated miR-24-3p replacement triggers apoptosis and autophagy, yielding robust tumor inhibition in xenografts [84]. While hepatocellular carcinoma efforts have focused on lipid systems, non-viral vectors including polymeric nanoparticles remain promising for miRNA delivery as cell-specific targeting and efficient transfection are optimized [80]. Beyond direct miRNA carriage, zein PNPs potentiate metformin’s antitumor activity while favorably reprogramming miRNA networks and the adenosine monophosphate-activated protein kinase (AMPK) axis in a breast cancer model, underscoring carrier-driven miRNA modulation in vivo [85]. Collectively, functionalized, stimuli-responsive PNPs offer a versatile miRNA delivery toolbox, with translational priorities centered on durability, biodistribution, and safety.

#### 4.2.3. miRNA-Based Cancer Therapeutics via Exosomes and Other Extracellular Vesicles

**Figure 1:**
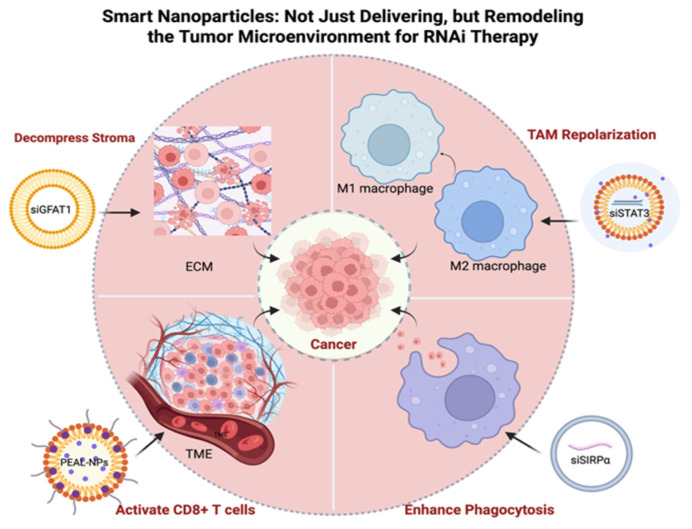
Smart Nanoparticles Enhance RNAi Therapy by Remodeling the Tumor Microenvironment. This schematic illustrates four key mechanisms by which nanoparticles overcome biological barriers, including: (1) Reprogramming TAMs from a pro-tumoral M2 phenotype to an anti-tumoral M1 phenotype; (2) Enhancing phagocytosis of cancer cells by blocking “don’t eat me” signals like SIRPα; (3) Activating adaptive immunity by promoting CD8+ T cell infiltration and anti-tumor activity; and (4) Decompressing the dense ECM to improve drug penetration. ECM, Extracellular Matrix; EV, Extracellular Vesicle; GFAT1, Glutamine-Fructose-6-Phosphate Aminotransferase 1; NP, Nanoparticle; PEAL, PEG-sheddable and Amino-Lipid-assisted; PNP, Polymer-based Nanoparticle; SIRPα, Signal Regulatory Protein Alpha; STAT3, Signal Transducer and Activator of Transcription 3; TAM, Tumor-Associated Macrophage; TME, Tumor Microenvironment. Created in BioRender. Zhang, C. (2026) https://BioRender.com/h9wt5g8, accessed on 30 September 2025.

Exosomes and other small extracellular vesicles are advancing miRNA therapeutics by combining intrinsic biocompatibility, immune evasion, and tissue tropism with engineerable surface ligands and payload control, thereby improving delivery across challenging barriers such as the BBB and hostile tumor microenvironments [33,86,87]. In lung cancer, LXY30 peptide–modified bone marrow mesenchymal stromal cells (MSCs) exosomes loaded with miR-30c, miR-181b, or miR-613 selectively targeted NSCLC, suppressing proliferation/migration and inducing apoptosis with in vivo safety, while underscoring the value of integrin-directed targeting [88]; conversely, Schwann cell–derived exosome miR-21-5p drives lung cancer growth and metastasis by repressing RECK, rationalizing anti–miR-21 or source-targeted interception strategies [89]. For glioblastoma, engineered exosomes packaging a polycistronic cassette encoding miR-124-2/miR-135a-2/let-7i achieved pan-subtype GSC inhibition and prolonged survival, outperforming single-miR or cocktail controls [90]. Immune engineering of T cell small extracellular vesicles (sEVs) with surface IL-2 reprograms vesicular miRNA content (e.g., miR-181a-3p/miR-223-3p) to downregulate tumor PD-L1, augment CD8+ T cell cytotoxicity, and synergize with anticancer drugs in melanoma [91]. In colorectal cancer, TM4SF5-targeted exosomes delivering miR-143 diminish metastasis-associated in colon cancer 1 (MACC1) signaling and curb invasion and tumor growth, whereas macrophage EVs enriched in NEAT1 promote colorectal cancer (CRC) stemness by sponging miR-34a-5p and restoring PEA15, highlighting both therapeutic cargo and decoy axes for intervention [92,93]. Prostate cancer bone pathology is shaped by PCa exosomal miR-92a-1-5p, which suppresses *COL1A1* to favor osteoclastogenesis, revealing a tractable EV-miRNA target in metastasis [94]. Translational infrastructure is coalescing: epithelial cell adhesion molecule (EpCAM)-aptamer nanoarrays with CRISPR/Cas13a quantify tumor-EV miR-21/23a in plasma, and urinary exosomal miR-16 supports rapid, low-cost prostate cancer screening—tools that can guide miRNA therapy selection and monitoring [87,95]. Finally, liver-programmed in vivo sEV biogenesis offers a scalable blueprint for RNA loading applicable to miRNA therapeutics [96], while TNBC- and MSC-EV-focused reviews map priorities in loading efficiency, ligand-guided targeting, GMP manufacture, and long-term safety [33,86]. The tumor microenvironment for RNAi therapy is shown in Figure 1. The details of RNAi-based therapeutic in various cancer are shown in Table 1.

## 5. Nanoparticles Used for the Delivery of RNAi-Based Therapeutics Against Cardiovascular Disease

### 5.1. siRNA-Based Therapeutics for Cardiovascular Disease

Liposomes and exosomes/extracellular vesicles (EVs) are propelling siRNA therapeutics for cardiovascular disease through precise, ligand-guided delivery to vascular and cardiac targets. In atherosclerosis, dual-targeted liposomes bearing anti-F4/80 and anti-CD68 antibodies concentrate Dll4 siRNA in M1 macrophages, suppressing Notch signaling, limiting vascular smooth muscle cell (VSMC) phenotypic switching/senescence, and reducing plaque vulnerability with favorable pharmacokinetics and safety [101]. Complementarily, VCAM-1-binding cationic liposomes deliver methylated NLRP3 siRNA to inflamed endothelium, blocking TNF-α-driven inflammasome/IL-1β signaling and LDL transcytosis; local and systemic dosing lowered endothelial LDL deposition and plaque burden in rodent models [102]. For ischemic heart disease, PEGylated cationic liposomes co-delivering apigenin and receptor for advanced glycation end products (RAGE) siRNA attenuated oxidative stress and inflammation via the RAGE/NF-κB axis, reducing arrhythmias, apoptosis, and infarct necrosis in vivo [103]. EVs add cardiac selectivity: small EVs engineered with a cardiac-targeting peptide (CTP-LAMP2b) enhanced myocardial uptake >2-fold and delivered siRAGE to dampen myocarditis inflammation in cells and mice [104]. Human serum–derived EVs functionalized with a fibroblast activation protein aptamer homed to injured myocardium; siTGFβ1 cargo reduced cardiac TGFβ1, fibrosis, and hypertrophy, improving function without systemic toxicity [105]. Together, liposomes (modular chemistry, co-delivery) and EVs (biocompatibility, peptide/aptamer retargeting) converge on macrophages, endothelium, and injured tissue to silence nodal pathways with promising translational profiles.

### 5.2. miRNA-Based Therapeutics for Cardiovascular Disease

Liposomes, extracellular vesicles and polymeric nanoparticles are advancing miRNA-based cardiotherapy by pairing disease-relevant targets with selective delivery and, when needed, small-molecule co-therapy. For atherosclerosis, multifunctional liposomes co-encapsulating EGCG and miR-223 simultaneously suppress oxidative/inflammatory pathways and upregulate ABCA1-driven lipid efflux, accumulate within plaques, and reduce foam cell formation in vivo [44]. Cardiac-targeted liposomes carrying an exosome-derived miR-185-5p inhibitor mitigate apoptosis and cuproptosis, improving left ventricular ejection fraction (LVEF) and limiting fibrosis in doxorubicin-induced dilated cardiomyopathy, illustrating organ-tropic formulation benefits [106]. Across pulmonary arterial hypertension, nanosystems—including liposomes and other nanocarriers—are highlighted for miRNA delivery to modulate pathogenic signaling beyond symptomatic vasodilation, underscoring translational potential and formulation choices by target biology [107]. EVs offer innate biocompatibility and cell-programmable cargo: cardiac-homing peptide–engineered bone marrow mesenchymal stem cell (BMSC) exosomes delivering miR-499a-5p blunt anthracycline cardiotoxicity via CD38/MAPK/NF-κB suppression [108]; human amniotic mesenchymal stem cell (hAMSC) exosomes transfer novel miRNA N-194 to endothelial cells, targeting ING5 to promote angiogenesis [109]; macrophage exosomal miR-204 mediates AT2 receptor–driven improvement of vascular calcification by targeting *RUNX2* and dampening Wnt/β-catenin signaling [110]. Therapeutic EV reprogramming can enhance efficacy, as depletion of deleterious miR-192-5p/miR-432-5p from progenitor cell sEVs augments post-infarction repair [111], whereas pathogenic adipose tissue–derived exosomal miR-132/212 accelerates atherogenesis but is mitigable (e.g., by melatonin), informing antimiR strategies [112]. Cardiac matrix–resident vesicles further reveal sex- and age-dependent miRNA synergies that shape anti-fibrotic responses and motivate rational miRNA combinations [113], while exosome RNA atlases in cardiac myxoma–related stroke provide diagnostic scaffolds for patient stratification [114]. Complementing EVs, polymeric nanoparticle–formulated antagomiR-21a-5p attenuates autoimmune myocarditis, reducing inflammation, fibrosis, and hypertrophy in vivo [115]. Collectively, carrier selection—liposomes for modular co-delivery, EVs for biocompatible, tissue-tropic transport, and polymeric nanoparticles for scalable miRNA delivery—enables precise miRNA modulation across cardiovascular indications. The details of RNAi-based therapeutic in cardiovascular disease are shown in Table 2.

## 6. Nanoparticles Used for the Delivery of RNAi-Based Therapeutics Against Respiratory Disease

### 6.1. siRNA-Based Therapeutics for Respiratory Diseases

Liposomes, polymeric nanoparticles, and extracellular vesicle-based systems are enabling organ- and cell-selective siRNA therapy in lung disease. In pulmonary fibrosis, intravenously administered liposomes passively accumulate in fibrotic foci to silence mechanosensitive ZNF416, dampen p-Smad2/3 signaling, and synergize with TGF-β receptor blockade to reduce injury and fibrosis [116], while *Sart1* siRNA liposomes reprogram macrophage polarization by limiting M2 infiltration and attenuate bleomycin-induced pathology [117]. For NSCLC perioperative care, an alginate hydrogel loaded with endoplasmic reticulum–modified liposomes co-delivering STAT3 siRNA and lidocaine induces tumor apoptosis, promotes M1 macrophages, decreases malignant pleural effusion, and relieves pain after a single application [37]. Polymeric oligopeptide-modified pBAE nanoparticles, anisamide-functionalized for tumor selectivity, protect and deliver mTOR siRNA with serum stability, achieving specific NSCLC knockdown and antitumor efficacy in vitro and in vivo [26]. EV-derived platforms broaden safety and targeting: edible, cation-free kiwi EVs carrying STAT3 siRNA and an EGFR aptamer suppress drug-resistant EGFR-mutant NSCLC with superior tolerability to cationic liposomes [69]; engineered EV–gold nanohybrids deliver B7-H4 siRNA to NSCLC cells and spheroids, sparing normal fibroblasts and inhibiting xenografts [70].

### 6.2. miRNA-Based Therapeutics for Respiratory Disease

Diverse nanocarriers are enabling precise miRNA therapeutics across respiratory indications. In idiopathic pulmonary fibrosis, inhalable liposomes delivering hsa-miR-30a-3p reverse myofibroblast programs and improve lung function, mirroring—but mechanistically focusing—the broader TGF-β attenuation seen with exosome therapy [118]. For chronic obstructive pulmonary disease (COPD), a technology landscape review highlights liposomes, polymeric and inorganic nanoparticles, dendrimers, and micelles as fit-for-purpose vehicles to deliver miRNA/antagomirs for immuno-inflammatory control and epithelial repair, emphasizing carrier–disease matching for biodistribution and compatibility [119]. In lung cancer, α3β1-integrin–targeted BMSC exosomes ferry miR-30c/181b/613 to suppress NSCLC growth, illustrating ligand-guided EV tropism [88], while Schwann cell exosomal miR-21-5p accelerates tumor progression, nominating antimiR-21 or EV-source interception as countermeasures [89]. Beyond oncology, dental pulp stem cell sEVs overexpressing miR-486 mitigate high-altitude pulmonary edema by modulating PTEN/PI3K/Akt/eNOS signaling [120], and neutrophil membrane–engineered Panax ginseng exosomes loaded with miR-182-5p alleviate septic acute lung injury via NOX4/Drp-1/NLRP3 pathway inhibition [121]. Notably, a synthetic-biology strategy that reprograms hepatocytes to self-assemble and export therapeutic siRNA within sEVs outperforms standard therapy for lung-metastatic osteosarcoma, providing a scalable in vivo EV-manufacturing blueprint readily adaptable to miRNA payloads for pulmonary delivery [96]. Together, inhalable liposomes and engineered EVs deliver disease-specific miRNA modulation with growing efficacy and translatability. The details of RNAi-based therapeutic in respiratory disease are shown in Table 3.

## 7. Nanoparticles Used for the Delivery of RNAi-Based Therapeutics Against Urological Disease

Synthetic nanoparticles and extracellular vesicles are enabling precise RNA therapeutics across the urologic spectrum. For siRNA delivery, tailored nanocarriers overcome nuclease instability and tissue penetration barriers in urological cancers—spanning polymeric and lipid nanoparticles, nanobubbles, and magnetic platforms—thereby supporting tumor-selective silencing strategies [123]. A representative therapeutic implementation uses layer-by-layer renal-targeted polymeric nanoparticles (PLGA core; chitosan/hyaluronan shells with a kidney-targeting peptide) to concentrate Arg-2 siRNA in proximal tubules after systemic dosing, attenuating oxidative/nitrosative stress, rescuing mitochondrial dysfunction, and reducing apoptosis in contrast-induced acute kidney injury models [27]. For miRNA modalities, EVs function as both carriers and disease informants: urinary exosomal miR-16-5p differentiates prostate cancer stages with practical, low-cost extraction, positioning uEVs for screening and treatment planning [95], while uEV miRNA signatures in diabetic kidney disease implicate apoptosis/inflammation pathways that can guide target selection and response monitoring [124]. Exosome cargo also reveals actionable biology—prostate cancer–derived exosomes transfer miR-92a-1-5p to suppress *COL1A1*, tipping bone homeostasis toward osteoclastogenesis and suggesting antimiR or EV-interception strategies against skeletal metastasis [94]. In crystal nephropathy, tubular cell–derived exosomes reprogram macrophage polarization via miRNA networks (e.g., miR-146a-5p, miR-200a-3p), reducing oxidative stress and calcium oxalate deposition in vivo, thereby nominating engineered EVs or miRNA cocktails for renoprotective therapy [125]. Together, advances in kidney-tropic polymeric systems and EV-based miRNA delivery/diagnostics converge on organ-specific biodistribution, pathway-level precision, and translational readiness in urologic disease. The details of RNAi-based therapeutic in urological disease are shown in Table 4.

## 8. Clinical Translation and Application

### 8.1. siRNA Therapeutics in Clinical Trials

Across early clinical development, siRNA therapeutics are being advanced with diverse delivery platforms that tailor biodistribution, exposure, and on-target engagement. A ligand-targeted cyclodextrin polymer nanocomplex (CALAA-01) packages an anti-R2 siRNA with PEG stabilization and transferrin targeting to transferrin-receptor–overexpressing tumors, with a first-in-human phase I defining safety, PK, immune readouts, and preliminary antitumor activity after IV dosing in refractory solid tumors [126]. For pancreatic adenocarcinoma, siG12D LODER uses an implantable local drug-eluting depot to maximize intratumoral exposure; an open-label phase 0/phase I program assesses distribution, DLTs/MTD, RP2D, and progression-free follow-up [127]. An intravenous siRNA regimen (TKM-080301) in advanced hepatocellular carcinoma employs a 3 + 3 dose-escalation with an expansion at the MTD, evaluating safety, PK, and preliminary efficacy with weekly infusions in 28-day cycles [128]. Biological nanoparticles are represented by mesenchymal stromal cell–derived exosomes loaded with *KRASG12D* siRNA (iExosomes), delivered IV on days 1, 4, and 10 in 14-day cycles, with endpoints spanning MTD/DLTs, PK, response, disease control, PFS, OS, and exploratory liquid/tissue biomarker assessments [129]. A cell-based approach infuses siRNA-transfected peripheral blood mononuclear cells (APN401) on days 1, 29, and 57 to characterize safety, immunologic effects, and clinical outcomes [130]. Extending beyond oncology, inhaled MBS-COV (SNS812) tests single and multiple ascending doses in healthy participants in a randomized, double-blind, placebo-controlled design to establish safety, tolerability, and PK for pulmonary delivery [131]. Collectively, these studies highlight the translational breadth of siRNA delivery—targeted nanoparticles, local depots, exosomes, cellular carriers, and inhalation—while converging on core phase I objectives: safety, MTD, PK, and early activity signals. A summary of siRNA therapeutics in clinical trials is shown in Table 5.

### 8.2. miRNA Therapeutics in Clinical Trials

**Figure 2:**
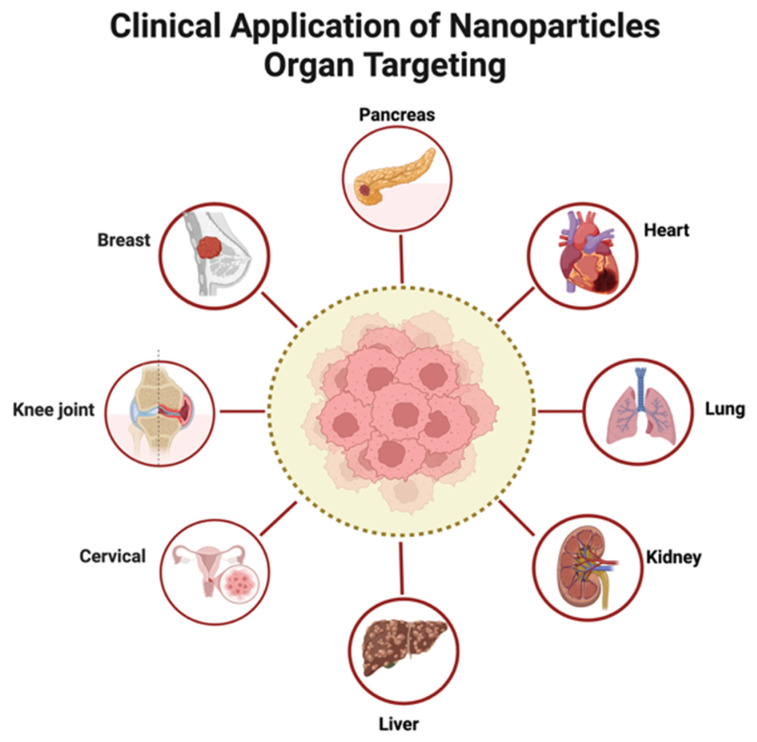
Clinical Application of Nanoparticles Organ Targeting. Created in BioRender. Zhang, C. (2026) https://BioRender.com/h9wt5g8, accessed on 30 September 2025.

Early clinical studies of miRNA therapeutics highlight diverse delivery strategies spanning nanoparticle carriers. In MesomiR-1, “TargomiRs”—EGFR-targeted, nonliving bacterial minicell nanoparticles encapsulating a miR-16 mimic—are administered intravenously in a phase I dose-escalation for recurrent malignant pleural mesothelioma and NSCLC to establish safety and the MTD via cohort-based escalation [132]. Cobomarsen (MRG-106), an inhibitor of oncogenic miR-155, uses flexible routes (intratumoral for cutaneous T-cell lymphoma (CTCL)/mycosis fungoides (MF); subcutaneous or intravenous systemically) across MF, chronic lymphocytic leukemia (CLL), diffuse large B-cell lymphoma (DLBCL), and adult T-cell leukemia/lymphoma (ATLL), with a multi-part design emphasizing safety, tolerability, pharmacokinetics, immunologic pharmacodynamics, and preliminary activity, employing a Days 1/3/5 lead-in followed by weekly dosing [133,134]. Beyond oncology, TenoMiR—a chemically synthesized, non-immunogenic miR-29a mimic engineered for improved stability, activity, and cellular uptake—targets tendon collagen remodeling in lateral epicondylitis in a global study building on a safe phase 1b, enabling treatment at initial diagnosis without invasive biopsies. MRX34, a liposomal microRNA product, is delivered intravenously in a phase I open-label dose-escalation (5 days on, 2 weeks off per 21-day cycle) to assess safety, pharmacokinetics, and pharmacodynamics in primary liver cancer and other advanced malignancies [135,136]. Collectively, these trials demonstrate clinical translation of miRNA therapy via distinct carriers—EGFR-targeted bacterial minicell nanoparticles, liposomal formulations, and chemically stabilized mimics/antagonists—tailored to disease biology while converging on core early-phase objectives: safety, dose finding, PK/PD characterization, and preliminary efficacy signals. Clinical applications of nanoparticle organ targeting are shown in Figure 2. A summary of miRNA therapeutics in clinical trials is shown in Table 6.

## 9. Approved siRNA-Based Therapies

A critical analysis of the current therapeutic landscape reveals that seven siRNA-based drugs have successfully transitioned from clinical trials to regulatory approval, validating RNAi as a powerful therapeutic modality. These approvals uniformly highlight the maturation of two key and distinct delivery platforms. The first, a LNP system for intravenous administration, is exemplified by Patisiran, which targets transthyretin (*TTR*) mRNA to treat hereditary transthyretin amyloidosis (hA*TTR*) [137]. In contrast, the subsequent approvals have all leveraged a successful carrier-free strategy: the direct conjugation of siRNA to a targeting ligand. This approach bypasses the need for a nanoparticle formulation for specific tissues. The most prominent example is the N-acetylgalactosamine (GalNAc)-siRNA conjugate platform. In this design, the siRNA payload is directly conjugated to a GalNAc ligand, which binds with high affinity to the asialoglycoprotein receptor (ASGPR) abundantly expressed on hepatocytes [138]. This interaction triggers rapid receptor-mediated endocytosis, leading to efficient delivery and potent gene silencing within the liver [139]. This highly effective GalNAc-conjugate platform has proven remarkably versatile, leading to the approval of Vutrisiran (targeting *TTR* for hA*TTR*) [140], Givosiran (targeting *ALAS1* for acute hepatic porphyria) [141], Lumasiran and Nedosiran (targeting *HAO1* and *LDHA*, respectively, for primary hyperoxaluria) [142,143], Inclisiran (targeting *PCSK9* for hypercholesterolemia) [144], and Fitusiran (targeting antithrombin for hemophilia A and B) [145]. The consistent success of these therapies underscores a common theme: all currently approved RNAi drugs target genes expressed in the liver, a direct consequence of the tropism of both LNP and GalNAc-based delivery systems. While no miRNA-based therapeutics have yet received approval, the clinical success of these seven siRNA drugs establishes a clear and effective blueprint for future RNAi drug development, demonstrating that targeted nanoparticle and conjugate systems can safely and effectively silence disease-causing genes in humans. The details of approved drugs are shown in Table 7.

## 10. Conclusions and Prospects

The convergence of RNAi-based mechanisms and advanced nanoparticle engineering has catalyzed a paradigm shift in precision medicine. As this review has detailed, the journey from the discovery of RNAi to its clinical application has been critically dependent on overcoming the formidable barriers of in vivo delivery. Liposomes, polymeric nanoparticles, and extracellular vesicles have emerged as the vanguard of this effort, each offering a unique combination of stability, biocompatibility, and targetability that has proven essential for translating the potential of siRNA and miRNA into tangible therapeutic strategies across oncology, cardiovascular disease, and beyond.

The studies highlighted herein demonstrate a clear trend toward sophisticated, multi-functional systems. We are moving beyond simple encapsulation to “smart” nanocarriers that feature ligand-guided targeting, stimuli-responsive release, and the capacity for rational co-delivery of both RNAi agents and small-molecule drugs. The use of biomimetic coatings (e.g., macrophage membranes) and endogenous carriers (e.g., exosomes) signifies a deeper integration with host biology, promising enhanced immune evasion and superior biodistribution. Furthermore, the ability to engineer these platforms to remodel the tumor microenvironment, reprogram immune cells, or navigate the blood–brain barrier illustrates the remarkable precision now within reach.

Despite this progress, the path to widespread clinical adoption requires addressing several key challenges. First, manufacturing, scalability, reproducibility and the associated regulatory hurdles remain a primary hurdle, particularly for complex biologic carriers like EVs. This challenge of scalability and reproducibility varies significantly across nanoparticle classes. For instance, lipid-based systems (e.g., LNPs) are the most mature, with scalable microfluidic manufacturing and well-defined release tests (e.g., particle size via DLS, encapsulation efficiency) that enabled the success of COVID-19 vaccines. In contrast, biogenic carriers like EVs present the highest barrier due to inherent variability from cellular sources, demanding distinct batch-to-batch metrics focused on particle concentration (via nanoparticle tracking analysis, NTA) and specific protein markers (e.g., CD9/CD63) to ensure consistency—a major challenge the field is actively working to overcome for potential MSC-derived exosome therapeutics. Establishing robust, GMP-compliant production methods is only the initial step. For regulatory approval and commercial viability, a comprehensive characterization, manufacturing, and controls (CMC) strategy is paramount. This involves defining stringent, quantifiable release criteria for identity, purity, and potency [29], as demanded by agencies like the food and drug administration(FDA) and european medicines agency (EMA). Identity must be rigorously confirmed using a multi-faceted approach, integrating qualitative analysis of specific protein markers (e.g., tetraspanins like CD9/CD63) and the demonstrated absence of co-isolating contaminants (e.g., lipoproteins, cytosolic proteins), alongside quantitative measurements of vesicle size, concentration, and morphology. Purity requires setting and meeting strict thresholds for process- and product-related impurities, including host cell DNA/proteins and endotoxins, often using metrics like the particle-to-protein ratio to ensure product integrity. Finally, a validated, function-based potency assay that reflects the therapeutic’s mechanism of action—such as measuring dose-dependent mRNA knockdown for an siRNA payload—is non-negotiable for ensuring batch-to-batch consistency and predicting clinical efficacy. Second, optimizing the therapeutic window continues to be a delicate balance. Enhancing on-target delivery while minimizing off-target effects and potential immunogenicity is an ongoing pursuit that demands further innovation in both carrier design and RNA sequence chemistry. A prime example of this challenge lies at the subcellular level: ensuring efficient endosomal escape. A crucial determinant of therapeutic success for RNAi nanoparticles is their ability to facilitate endosomal escape, releasing the siRNA payload into the cytoplasm to engage the RNA-induced silencing complex (RISC). Several distinct strategies have been developed to overcome this barrier, each with a growing body of in vivo evidence. The most clinically advanced strategy involves ionizable lipids, which are central to the success of FDA-approved lipid nanoparticles (LNPs) like Patisiran (Onpattro^®^) [137,147]. These lipids are engineered with an apparent pKa that keeps them neutral at physiological pH (≈7.4), but upon endosomal acidification (pH ≈ 5.0–6.5), they become protonated, disrupting the membrane and leading to payload release. Another widely explored chemical strategy is the “proton sponge” effect, commonly associated with cationic polymers like polyethyleneimine (PEI), which uses osmotic pressure to rupture the endosome [13,148]. More biomimetic approaches include using fusogenic lipids or peptides that merge with the endosomal membrane [149], or even rationally designed “mini-proteins” that unfold at low pH to expose membrane-disrupting domains [12]. Finally, physical triggers like light or ultrasound offer external control over release, with preclinical studies demonstrating significant enhancement of targeted gene silencing in vivo [150,151]. While effective, these strategies must be carefully refined to maximize cytoplasmic delivery without inducing cytotoxicity, illustrating the fine line between efficacy and safety. Third, the long-term safety profile of these nanoparticles, including their accumulation and degradation in vivo, must be rigorously characterized to ensure patient safety over chronic treatment courses.

Looking forward, the future of nanoparticle-mediated RNAi delivery is marked by several promising research trajectories. We anticipate the rise of in vivo-generated therapeutics, where strategies like the hepatocyte-reprogramming system create a “bioreactor” within the patient to produce a sustained supply of therapeutic EVs. The integration of artificial intelligence and machine learning will likely accelerate the discovery of novel nanoparticle formulations and predict their in vivo behavior, offering a potential pathway for high-throughput screening of carrier candidates. Finally, the synergy between diagnostic and therapeutic (theranostic) platforms, where nanoparticles not only deliver a payload but also provide real-time feedback on target engagement and therapeutic response, will pave the way for more precise and personalized RNAi medicine. By continuing to bridge the gap between materials science, molecular biology, and clinical medicine, nanoparticle-based RNAi delivery is poised to unlock a new class of therapeutics with the potential to address humanity’s most challenging diseases.

## Figures and Tables

**Table 1:** RNAi-based therapeutic delivery technologies in various cancer.

Target Gene(s)	Delivery Vehicle	Administration Method	Delivery Site (Tissue/Organ)	Disease/Model	Rats or Mice	RNAi Type	Key Outcomes/Efficacy	Ref.
BCL2L1/BFL-1	Cationic liposome + F-PEI polymer (co-delivering PROTAC DT2216)	Not specified (likely systemic)	Malignant Tumors (General)	Malignant Tumor Xenograft	*Mice*	siRNA	“Remarkable degradation” of target proteins and inhibition of tumor cells in vivo and in vitro.	[48]
MEF2D	tLyp-1 peptide-modified liposome (co-delivering SN38)	Systemic	HCC	HCC (H22 cells)	*Mice*	siRNA	Reduced PD-L1 expression, suppressed tumor growth, and increased CD4+ T cells in the tumor.	[38]
TWF1	BRBP1 peptide-modified liposome (co-delivering Paclitaxel)	Systemic	Brain metastases	Brain Metastatic Breast Cancer (231-BR cells)	*Mice*	siRNA	Crossed BBB, accumulated in brain metastases, inhibited tumor growth, and reversed Paclitaxel resistance.	[97]
STAT3	Endoplasmic reticulum-modified liposome in a hydrogel (co-delivering Lidocaine)	Intra-operative injection	Lung resection surface	Orthotopic NSCLC	*Mice*	siRNA	Inhibited tumor growth, reduced MPE volume, and provided pain relief with a single administration.	[37]
Midkine (MDK)	Polymer-locking fusogenic liposome (Plofsome)	Systemic	Brain Tumor	Orthotopic GBM	*Mice*	siRNA & CRISPR-Cas9	Crossed BBB, reduced TMZ resistance, and significantly inhibited GBM growth.	[42]
HPV16 E6	PEGylated cationic liposome	Not specified (in vitro study)	Cervical Cancer Cells	Cervical Cancer (CaSki cells)	In vivo	siRNA	Reduced HPV16 E6 protein levels, restored p53, and inhibited proliferation (25.7%), migration (95.7%), and invasion (97.8%).	[98]
GFAT1	Macrophage membrane-camouflaged, LFC131-targeted liposome	Systemic	Tumor	PDAC	Not specified	siRNA	Reduced HA levels, improved permeability of chemotherapy, and demonstrated potent antitumor efficacy with Doxil.	[47]
PLK1	Folate-modified PEGylated cationic liposome	Systemic	Tumor	Nasopharyngeal Carcinoma Xenograft (KB cells)	*Mice*	siRNA	Achieved selective tumor cell growth inhibition and accumulation of siRNA in tumor xenografts.	[99]
BIRC5 (Survivin)	Dermatan sulfate/chitosan-graft-poly(methyl methacrylate) NPs	Not specified (in vitro)	Tumor cells	Triple-Negative Breast Cancer (4T1 cells)	In vivo	siRNA	Silenced survivin; inhibited cell viability, migration, and proliferation in vitro.	[57]
HIF-1α	Hyaluronic acid-displaying chitosan NPs	Systemic (in vivo)	Pancreatic tumor cells	Pancreatic Cancer (multiple cell lines)	In vivo	siRNA	Significant knockdown of HIF-1α and downstream genes in vivo. HMW chitosan was more effective for knockdown.	[63]
AFP	Polymeric NPs (co-administered with Sunitinib or Sorafenib)	Not specified (in vitro)	Tumor cells	HCC (cell model)	In vivo	siRNA	With Sunitinib, AFP-mRNA expression was decreased to ~28.5%. Cell viability reduced to ~39% (with Sorafenib) and ~44% (with Sunitinib).	[56]
*cPLA2*	Blood exosomes (co-delivering Metformin)	Systemic	GBM	Patient-Derived Xenograft GBM model	*Mice*	siRNA	Crossed BBB, impaired tumor energy metabolism, reduced tumor growth, and prolonged survival.	[71]
IGF1 & IGF1R (via miR-603 delivery)	PR_b peptide-functionalized PEGylated liposome with a PEI/miRNA core	Not specified (in vitro)	Patient-derived glioblastoma stem-like cells	GBM	In vivo	miRNA	22-fold increase in intracellular miR-603 levels; decreased IGF1/IGF1R expression; sensitized glioblastoma cells to ionizing radiation.	[100]
NF-κB, miR-191-5p (inhibition) & p53, miR-543 (upregulation)	Zein polymeric nanoparticles (delivering Metformin, not RNAi)	Not specified (in vivo)	Solid Tumor	Ehrlich Carcinoma (solid tumor in mice)	*Mice*	miRNA (downstream effect)	This study delivered Metformin. The observed miRNA changes were a result of the drug’s action, not the primary cargo. Produced a pronounced anticancer effect.	[85]
MACC1 (via miR-143)	Adipose-derived stem cell exosomes targeting TM4SF5 protein	Not specified (in vivo)	Tumor	Colorectal Cancer xenograft model (HCT116 cells)	*Mice*	miRNA	Resulted in the smallest tumor size and lowest growth rate compared to controls (*p* < 0.05); decreased MACC1 expression.	[92]

Abbreviations: siRNA, short interfering RNA; PROTAC, proteolysis targeting chimera; HCC, Hepatocellular Carcinoma; BBB, blood–brain barrier; NSCLC, non-small cell lung cancer; MPE, malignant pleural effusion; HA, hyaluronic acid; PDAC, pancreatic ductal adenocarcinoma; HCC, hepatocellular carcinoma; HMW, high molecular weight; GBM, Glioblastoma; IGF1/IGF1R, Insulin-Like Growth Factor 1/Insulin-Like Growth Factor 1 Receptor; miRNA, micro RNA; MACC1, metastasis-associated in colon cancer 1.

**Table 2:** RNAi-based therapeutic delivery technologies in cardiovascular disease.

Target Gene(s)	Delivery Vehicle	Administration Method	Delivery Site (Tissue/Organ)	Disease/Model	Rats or Mice	RNAi Type	Key Outcomes/Efficacy	Ref.
NLRP3	VCAM-1 peptide-targeted cationic liposomes	Local (carotid) & Systemic (IV)	Vascular Endothelium	Atherosclerosis (Rat partial carotid ligation & ApoE-/- mice)	*Mice*	siRNA	Attenuated LDL deposition in the carotid artery (rat model); reduced plaque formation (mouse model).	[102]
TGFβ1	Human serum-derived extracellular vesicles (hEVs) functionalized with FAP aptamer	IV	Injured Cardiac Tissue	Cardiac Injury (Ang II-treated mice)	*Mice*	siRNA	Significantly improved cardiac function, reduced myocardial fibrosis, and decreased cardiomyocyte cross-sectional area (*p* < 0.05) without systemic toxicity.	[105]
miR-185-5p (inhibition)	Cardiac-targeting peptide-functionalized liposomes	Not specified (in vivo)	Cardiac tissue	Dilated Cardiomyopathy (DOX-induced mice)	*Mice*	miRNA (inhibitor)	Increased LVEF by 27.3%; reduced myocardial fibrosis by 36.5%; enhanced survival. Reduced apoptosis by 42.6% in vitro.	[106]
CD38 (via miRNA-499a-5p)	Cardiac Homing Peptide-engineered bone marrow MSC-derived exosomes	Not specified (in vivo)	Heart	Cardiotoxicity (DOX-induced model)	*Mice*	miRNA	Significantly improved electrocardiogram, decreased myocardial enzymes, and improved cardiac pathological changes.	[108]

Abbreviations: VCAM, vascular cell adhesion molecule; siRNA, short interfering RNA; FAP, fibroblast activation protein; IV, Intravenous; DOX, doxorubicin; LVEF, left ventricular ejection fraction; MSC, mesenchymal stem cell; miRNA, microRNA.

**Table 3:** RNAi-based therapeutic delivery technologies in respiratory disease.

Target Gene(s)	Delivery Vehicle	Administration Method	Delivery Site (Tissue/Organ)	Disease/Model	Rats or Mice	RNAi Type	Key Outcomes/Efficacy	Ref.
ZNF416	Liposomes	Tail-vein injection	Lung (fibrotic area)	Pulmonary Fibrosis (Silica- or Bleomycin-induced mice)	*Mice*	siRNA	Co-administration with SB431542 significantly protected mice against lung injury and fibrosis. Passively targeted the fibrotic lung area.	[116]
*Sart1*	Liposomes	Intratracheal	Lung (macrophages)	Pulmonary Fibrosis (Bleomycin-induced mice)	*Mice*	siRNA	Significantly protected mice against lung injury and fibrosis by attenuating M2 macrophage infiltration.	[117]
*Smad4*	Exosome membrane-DOTAP hybrid nanoscaffolds (DOTAP/si*Smad4*@EM)	Pulmonary	Lung (pulmonary fibroblasts)	Idiopathic Pulmonary Fibrosis (Mouse model)	*Mice*	siRNA	Significantly down-regulated *Smad4* expression with augmented anti-fibrosis efficiency and excellent biocompatibility.	[122]
CNPY2/PERK/DDIT3 (via miR-30a-3p)	Inhalable liposomes	Dry powder inhalation	Lung (myofibroblasts)	Idiopathic Pulmonary Fibrosis (Bleomycin-induced mice)	*Mice*	miRNA (mimic)	Consistently improved pulmonary function across six tests; promoted de-differentiation of profibrotic myofibroblasts.	[118]

Abbreviations: siRNA, short interfering RNA; DOTAP, 1,2-Dioleoyl-3-trimethylammonium-propane chloride; CNPY2, canopy FGF Signaling Regulator 2; PERK, protein kinase R-Like endoplasmic reticulum kinase; DDIT3, DNA Damage-Inducible Transcript 3; miRNA, microRNA.

**Table 4:** RNAi-based therapeutic delivery technologies in urological disease.

Target Gene(s)	Delivery Vehicle	Administration Method	Delivery Site (Tissue/Organ)	Disease/Model	Rats or Mice	RNAi Type	Key Outcomes/Efficacy	Ref.
Arginase-2	KTP-modified, layer-by-layer polymeric nanoparticles	Tail-vein injection	Kidneys (proximal tubular cells)	CI-AKI (Iohexol-induced mice)	*Mice*	siRNA	Alleviated oxidative stress, rescued mitochondrial dysfunction, and reduced apoptosis, demonstrating a robust therapeutic effect.	[27]
Multiple (via miRNA delivery)	Exosomes from healthy HK2 cells (kidney epithelial cells)	Renal subcapsular injection	Kidney	CaOx Crystal-Induced Kidney Injury	*Rats*	miRNA	Effectively reduced CaOx crystal deposition and tubular damage; suppressed oxidative stress and M1 macrophage polarization.	[125]
*COL1A1* (via miRNA-92a-1-5p)	Prostate cancer cell-derived exosomes	Not specified (in vivo)	Bone	In vivo osteolysis model	*Mice*	miRNA	Induced osteolysis and promoted osteoclast differentiation in vivo.	[109]

Abbreviations: KTP, kidney targeting peptide; CI-AKI, contrast-induced acute kidney injury; siRNA, short interfering RNA; CaOx, Calcium Oxalate; miRNA, microRNA.

**Table 5:** Organ-targeted siRNA drug delivery studied in clinical trials.

Drug Name	Genetic/Protein Target	Delivery Vehicle	Administration Method	Tissue	Disease	ClinicalTrials.gov Identifier	Status	Ref.
CALAA-01	*RRM2*	Cyclodextrin nanoparticles	Intravenous infusion	Tumor	Advanced solid tumors	NCT00689065	Phase I (Terminated 2013)	[126]
*EphA2*-targeting DOPC	*EphA2*	Neutral liposome	administered intravenously	Tumor	Advanced Malignant Solid Neoplasm	NCT01591356	Phase I (Active, not recruiting2025-07)	[127]
TKM-080301	PLK1	LNPs	Intravenously	Liver	Hepatocellular carcinoma	NCT02191878	Completed (2019-01)	[128]
MSC-derived Exosomes with KRAS G12D siRNA	*KrasG12D*	MSC-derived exosomes	Intravenously	Pancreas	Pancreatic cancer	NCT03608631	Phase 1 (Active, not recruiting)	[129]
APN401	Cbl-b	siRNA-transfected Peripheral Blood Mononuclear Cells	Intravenously	Tumor	Solid tumors	NCT03087591	Completed (2024-10)	[130]
NU-0129	*Bcl2L12*	SNA platform	Intravenously	Solid Tumors/Immune cells	Glioblastoma multiforme	NCT03020017	Completed (2022-08)	ND
MBS-COV	SNS812	ND	Inhalation	Brain	SARS-CoV-2	NCT05677893	Completed (2025-03)	[131]

Abbreviations: MSC, mesenchymal stem cell; SNA, spherical nucleic acid; ND, not disclosed.

**Table 6:** Organ-targeted miRNA drug delivery studied in clinical trials.

Drug Name	Genetic/Protein Target	Delivery Vehicle	Administration Method	Tissue	Disease	ClinicalTrials.gov Identifier	Status	Ref.
TargomiRs	miR-16 Mimic	EnGeneIC Delivery Vehicle	IV injected	Lung/Pleura	Malignant Pleural Mesothelioma; Non-Small Cell Lung Cancer	NCT02369198	Phase 1 Completed in 2017	[132]
Obefazimod (ABX464)	miRNA-124	ND	Oral	Colon/Intestines	Ulcerative Colitis	NCT05507203 NCT05507216	Phase 3 (Completed in 2025-08)	ND
cobomarsen (MRG-106)	miR-155	LNA-mediated	Subcutaneous intravenous	Blood/Lymphoid tissue	ATLL	NCT02580552 NCT03713320 NCT03837457	Phase I (Completed-2020) Phase II (Terminated-2020) Phase II (Terminated-2020)	[133,134]
TenoMiR	miR-29a	Lateral epicondylitis	Injection	Tendon	Single intralesional injection	NCT06192927	Phase II (Completed-2025)	ND
MRX34	miR-RX34	liposomal	intravenously	Liver	Primary Liver Cancer	NCT01829971 NCT02862145	Phase I (Terminated-2016)	[135,136]

Abbreviations: ATLL, adult T-cell leukemia/lymphoma; ND not disclosed.

**Table 7:** Approved siRNA-based therapeutics.

Trade Name	Target Gene	Delivery Vehicle	Route	Tissue/Organ	Disease	Date of Approved	Clinical Trial ID	Refs.
Patisiran (Onpattro^®^)	*TTR*	LNP	IV	Liver	hA*TTR*	2018-08	NCT01960348	[137]
Vutrisiran (Amvuttra^®^)	*TTR*	GalNAc	SI	Liver	hA*TTR*	2022-06	NCT03759379	[140]
Givosiran (Givlaari^®^)	*ALAS1*	GalNAc	SI	Liver	AHP	2019-11	NCT03338816	[141]
Lumasiran (Oxlumo^®^)	*HAO1*	GalNAc	SI	Liver	PH1	2020-11	NCT03681184	[142]
Nedosiran (Rivfloza™)	*LDHA*	GalNAc	SI	Liver	PH1	2023-09	NCT03847909	[143]
Inclisiran (Leqvio^®^)	*PCSK9*	GalNAc	SI	Liver	Hypercholesterolemia	2021-12	NCT03399370, NCT03400800	[144]
Fitusiran	*AT*	GalNAc	SI	Liver	Hemophilia A and B	2025-13	NCT03417102, NCT03417245	[145,146]

Abbreviations: *TTR*, Transthyretin; *ALAS1*, Aminolevulinate Synthase 1; *HAO1*, Hydroxyacid Oxidase 1; *LDHA*, Lactate Dehydrogenase A; *PCSK9*, Proprotein Convertase Subtilisin/Kexin type 9; *AT*, Antithrombin; LNP, Lipid Nanoparticle; GalNAc, N-Acetyl-Galactosamine; IV, Intravenous Infusion; SI, Subcutaneous Injection; AHP, Acute Hepatic Porphyria; PH1, Primary Hyperoxaluria type 1.

## Data Availability

No new data were created or analyzed in this study. Data sharing is not applicable to this article.

## Abbreviations

The following abbreviations are used in this manuscript:

RNAi	RNA interference
miRNA	microRNA
siRNA	small interfering RNA
MTD	maximum tolerated dose
PK/PD	pharmacokinetics/pharmacodynamics
GMP	good manufacturing practice
RISC	RNA-induced silencing complex
RES	reticuloendothelial system
CARPA	complement activation-related pseudoallergy
PRRs	pattern recognition receptors
RIG-I	retinoic acid-inducible gene I
MDA5	melanoma differentiation-associated gene 5
NADs	nucleic acid drugs
CRISPR	clustered regularly interspaced short palindromic repeats
LNPs	lipid nanoparticles
BBB	blood–brain barrier
NSCLC	non-small cell lung cancer
ROS	reactive oxygen species
RNPs	ribonucleoprotein complexes
BRBP1	brown rice bran powder1
EMT	epithelial-mesenchymal transition
PROTACs	proteolysis targeting chimeras
PNPs	polymeric nanoparticles
ECM	extracellular matrix
HCC	hepatocellular carcinoma
TNBC	triple-negative breast cancer
PEG	polyethylene glycol
VEGF	vascular endothelial growth factor
PEAL	mPEG-PLGA-PLL
PDAC	pancreatic ductal adenocarcinoma
TME	tumor microenvironment
PEI/PPI	polyethylenimine/polypropylenimine
TAMs	tumor-associated macrophages
EGFR	epidermal growth factor receptor
cPLA2	cytoplasmic phospholipase A2
PDX	patient-derived xenograft
PTRF	polymerase 1 and transcript release factor
PPM1D	mutation of p53-induced protein phosphatase 1
DIPG	diffuse intrinsic pontine gliomas
DARS-AS1	delivery of aspartyl-tRNA synthetase-antisense RNA 1
MAPK/ERK	mitogen-activated protein kinase/extracellular signal-regulated kinase
HCR	hybridization chain reaction
TPP	triphenylphosphine
AMPK	5′-adenosine monophosphate-activated protein kinase
MSCs	mesenchymal stromal cells
GSC	glioblastoma stem cell
MACC1	metastasis-associated in colon cancer 1
CRC	colorectal cancer
EpCAM	epithelial cell adhesion molecule
RAGE	receptor for advanced glycation end products
EGCG	Co-encapsulating epigallocatechin-3-gallate
LVEF	left ventricular ejection fraction
BMSC	bone marrow mesenchymal stem cell
hAMSC	human Amniotic Mesenchymal Stem Cell
VSMC	vascular smooth muscle cell
COPD	chronic obstructive pulmonary disease
DLTs	dose-limiting toxicities
RP2D	recommended phase 2 dose
CTCL/MF	cutaneous T-cell lymphoma/mycosis fungoides
CLL	chronic lymphocytic leukemia
DLBCL	diffuse large B-cell lymphoma
ATLL	adult T-cell leukemia/lymphoma
NTA	nanoparticle tracking analysis
